# Visfatin and Subclinical Atherosclerosis in Type 2 Diabetes: Impact of Cardiovascular Drugs

**DOI:** 10.3390/medicina59071324

**Published:** 2023-07-18

**Authors:** Kati Kärberg, Alastair Forbes, Margus Lember

**Affiliations:** 1 Institute of Clinical Medicine, University of Tartu, L. Puusepa 8, 50406 Tartu, Estonia; alastair.forbes@ut.ee (A.F.); margus.lember@ut.ee (M.L.); 2 Internal Medicine Clinic, Tartu University Hospital, L. Puusepa 8, 50406 Tartu, Estonia

**Keywords:** adipokines, atherosclerosis, type 2 diabetes mellitus, intima–media thickness, ankle–brachial index, cardiovascular drugs

## Abstract

*Background and Objectives*: The role of adipokines in the development of atherosclerosis in type 2 diabetes (T2DM) has not yet been fully elucidated. The effects of drugs on adipokine concentrations have only been evaluated in very few studies, although they may be of clinical importance. This study aimed to assess whether the concentrations of circulating adipokines could predict subclinical atherosclerosis in patients with T2DM, as well as their interactions with commonly used cardiovascular drugs. *Materials and Methods*: Our population-based cross-sectional multicentric study included 216 participants with T2DM but without previously diagnosed atherosclerosis. The carotid artery intima–media thickness (IMT), plaque and ankle–brachial index (ABI) metrics were measured. Resistin, visfatin, retinol-binding protein 4, high molecular weight adiponectin and leptin levels were evaluated using Luminex’s xMAP technology. *Results*: Visfatin and resistin concentrations correlated positively with IMT (*p* = 0.002 and *p* = 0.009, respectively). The correlation of visfatin to IMT ≥ 1.0 mm was significant in males (*p* < 0.001). Visfatin had a positive correlation with IMT ≥ 1.0 mm or plaque (*p* = 0.008) but resistin only correlated with plaque (*p* = 0.049). Visfatin predicted IMT ≥ 1.0 mm or plaque in patients on β-blocker monotherapy (*p* = 0.031). Visfatin lost its ability to predict subclinical atherosclerosis in patients taking angiotensin-converting enzyme inhibitors, angiotensin receptor blockers, calcium channel blockers or statins. After adjustments for risk factors for atherosclerosis and cardiovascular drugs, visfatin maintained an independent association with mean IMT (*p* = 0.003), IMT ≥ 1.0 mm or plaque (*p* = 0.005) and ABI ≤ 0.9 (*p* = 0.029). *Conclusions:* Visfatin could be used as a marker of subclinical atherosclerosis in patients with T2DM, especially in males. The assessment of visfatin concentration could aid in identifying individuals who could benefit from implementing preventive measures against atherosclerosis.

## 1. Introduction

Diabetes is one of the fastest growing global health challenges [1]. There is a strong link between the increased prevalence of overweight and obesity and type 2 diabetes (T2DM). In Europe in 2021, the prevalence of overweight was 36% and that of obesity was 29% [1,2]. By 2030, approximately 215 million Europeans could be at risk of complications from obesity and 67 million people are predicted to develop diabetes [2].

Obesity is a chronic, complex, multifactorial disease that is defined by excessive adiposity [3]. Although adipose tissue was first recognised as an endocrine organ almost four decades ago, its function, dysfunction and interactions with other systems and chronic diseases that affect them, including cardiovascular diseases, are still not completely clear [4].

Adipose tissue-derived factors, known as adipokines (such as adiponectin, leptin, resistin, retinol-binding protein 4 (RBP-4) and visfatin), influence atherosclerosis development and can have either protective or aggravating effects [5].

Adiponectin is considered to be anti-atherogenic as it regulates lipid metabolism and insulin sensitivity. It is inversely associated with subclinical atherosclerosis [6].

Although leptin’s primary function is to regulate body weight in the long term, leptin resistance occurs in obesity and its concentrations correlate positively with the lipid markers of inflammation and atherosclerosis in asymptomatic T2DM [7]. Resistin is thought to have multiple roles in vascular inflammation, lipid accumulation and plaque destabilisation and its levels correlate positively with various cardiovascular disorders [8]. RBP-4 has been proposed as a marker of endothelial dysfunction for newly diagnosed T2DM as it correlates positively with carotid atherosclerosis [9].

To date, the fewest studies have been carried out on visfatin, which seems to behave as an aggravating adipokine [10]. Higher visfatin levels have been described in T2DM, obesity and subclinical atherosclerosis, but they have mostly been reported in advanced atherosclerosis [8,11,12,13,14,15,16,17].

The fact that atherosclerosis progresses faster in diabetes is well known, but the potential association of subclinical atherosclerosis with (newer) adipokines has been poorly described. There is currently a lack of studies justifying the routine measurement of adipokines when screening for the development of early diabetic complications in patients with T2DM who already receive cardiovascular-oriented combination treatments. Moreover, the effects of medication use on the circulating levels of adipokines have generally only been described one drug group at a time [18,19,20,21,22]. There are surprisingly few studies describing changes in adipokine concentrations and their predictive power for subclinical atherosclerotic disease when linked to drug use in type 2 diabetes [11,18].

Therefore, the present study aimed to assess whether the circulating levels of adipokines could predict subclinical atherosclerosis in patients with type 2 diabetes and to what extent each effect was influenced by commonly used cardiovascular drugs.

## 2. Materials and Methods

### 2.1. Population

This population-based cross-sectional multicentric study included 216 participants with T2DM, who were recruited from 13 general practices in Estonia from November 2014 to March 2017. T2DM was defined according to the World Health Organization 2006/2011 recommendations as fasting plasma glucose ≥ 7.0 mmol/L (126 mg/dL), 2-h plasma glucose ≥ 11.1 mmol/L (200 mg/dL) or haemoglobin A1c (HbA1c) ≥ 6.5% (48 mmol/mol) [1]. The study included patients (aged 30–70 years) who were able to visit outpatient clinics independently, had never been diagnosed with atherosclerosis or its complications and weighed less than 140 kg (the upper limit of the dual-energy X-ray absorptiometer). Our exclusion criteria were the other types of diabetes, pregnancy or lactation, coronary artery, carotid artery, peripheral artery or cerebrovascular diseases, a history of chronic inflammatory diseases, malignant tumours or other severe diseases.

The study was conducted in accordance with the Declaration of Helsinki and its amendments and was approved by the Research Ethics Committee of the University of Tartu (protocol number: 223/T-17; 25 February 2013). Each study participant provided informed consent in writing. All patient details were anonymised.

### 2.2. Clinical and Biochemical Measurements

The subjects were invited to morning appointments after at least 10 h of fasting. All subjects provided written informed consent before their individual interviews. Data regarding medical history, smoking habits and current medication were collected via a non-validated questionnaire. The participants’ medical histories and their current use of prescription medications were verified using the national electronic health information and prescription system by a general practitioner. Antiplatelet and anticoagulant therapies were not included in the analysis as they are also used for ischemic complications and antiplatelet therapy is not controlled in Estonia (i.e., it is available to buy without prescription). Only pharmacy-dispensed medications were considered.

The height, waist circumference and weight of the participants were measured to the nearest 0.1 cm, 0.1 cm and 0.1 kg, respectively. Their body mass index (BMI) was then calculated as their weight in kilograms divided by the square of their height in metres (kg/m^2^).

Blood pressure measurements were obtained from each patient in the seated position using a standard mercury sphygmomanometer. The average of three measurements was used for the statistical analysis.

Venous blood samples were obtained to measure HbA1c, total cholesterol, triglycerides, low-density lipoprotein cholesterol, high-density lipoprotein cholesterol (HDL-C) and high-sensitivity C reactive protein levels (routine laboratory methods). Non-HDL-C was then calculated from the lipid profiles (total cholesterol minus HDL-C).

Luminex’s xMAP technology was used to determine the serum levels of high molecular weight (a large multimer of 12–18 subunits) adiponectin, leptin, RBP-4, resistin and visfatin.

Hypertension was defined as systolic blood pressure ≥ 140 mmHg and/or diastolic blood pressure ≥ 90 mmHg, having a history of hypertension or current treatment with antihypertensive drugs, hyperlipidaemia from low-density lipoprotein cholesterol levels ≥ 2.6 mmol/L (100 mg/dL) (as diabetic patients without target organ damage have moderate cardiovascular risks) or the use of statins [23].

The International Physical Activity Questionnaire (short form) was used to assess the subjects’ appropriate individual physical activity. Activity was calculated as a continuous variable (i.e., the metabolic equivalent of weekly task minutes) [24]. The reporting of this study conformed to the STROBE guidelines [25].

### 2.3. Measurement of Carotid Artery Intima–Media Thickness

Carotid intima–media thickness (IMT) was used to characterise subclinical atherosclerosis in the carotid artery. A single sonographer performed the measurements using a high-resolution B-mode tomographic ultrasound system (Philips Affiniti 70 G, Philips Healthcare, 3000 Minuteman Road, Andover, MA, USA) with a linear 12 MHz transducer, as described previously [26]. For an overview of the vessel orientation, plaque and surrounding structures, a transverse scan was taken of the proximal part of the common carotid artery (CCA) throughout the bulb to the distal internal carotid artery (ICA) and the external carotid artery. Longitudinal scanning techniques were used to create two distinct parallel echogenic lines representing the intima and media layers. Measurements were taken from anterior, lateral and posterior imaging planes in the CCA (10 mm proximal), bulb and ICA (5–10 mm from the bulb) in plaque-free areas. In each section of the carotid artery, three measurements were taken manually at a lower resolution (i.e., the zoom function was not used) from the far wall on both sides of the artery (54 measurements per patient). Images and cine-loops were obtained using a three-lead electrocardiogram and saved as dynamic sequences using Digital Imaging and Communications in Medicine (DICOM) for offline analysis with a RadiAnt DICOM Viewer. All recordings were reviewed and measured by the same expert. The intra-observer variability for measuring IMT was 3.5% (according to Bland–Altman analysis).

In accordance with the American Society of Echocardiography and Mannheim Carotid Intima–Media Thickness and Plaque consensus statement, subclinical atherosclerosis was defined as IMT ≥ 1 mm and plaque was defined as focal wall thickening that was at least 50% greater than the surroundings or as focal regions with IMT greater than 1.5 mm [27,28]. The presence of plaque was recorded on a yes or no basis. Atherosclerosis was defined as IMT ≥ 1 mm or the presence of plaque.

### 2.4. Measurement of the Ankle–Brachial Index

The ankle–brachial index (ABI) was used to characterise peripheral arterial atherosclerosis. The technique described in the American Heart Association’s statement was used for the measurements [29]. An Atys Microflow S 8 MHz Doppler device (Atys Medical, 17, Parc d’Arbora, 69510, Soucieu en Jarrest, France) was used to locate the arterial pulse and then two systolic pressure measurements were obtained from each arm and ankle, with the average being recorded for both sides. To calculate the ABI, the highest ankle blood pressure (dorsalis pedis or posterior tibial artery) was divided by the highest arm blood pressure (right or left). For statistical analysis, we used the highest ABI for each side. ABI values less than 0.9 defined peripheral artery disease.

### 2.5. Measurement of the Android to Gynoid Ratio

A Lunar Prodigy Advance dual-energy X-ray absorptiometry machine (GE Healthcare, Waukesha, WI, USA) was used to evaluate the percentages of android and gynoid fat. All scans were performed in accordance with the manufacturer recommended positioning and measurements were carried out by a qualified and experienced technician. The regions of interest for regional body composition were defined using the software provided by the manufacturer. The android to gynoid fat ratio (A/G) was calculated as android fat divided by gynoid fat. An A/G ratio of more than 1 was defined as a risk factor.

### 2.6. Statistical Analysis

The sample size was calculated with a margin of error of 0.05, a population size of 1.329 million and a population proportion of interest of 8%. The following formulae were used for these calculations:

n = [z^2^x Φ(1 − Φ)]/ε^2^

n′ = n/{1 + [ z^2^x Φ(1 − Φ)]/ε^2^N}

where z is the z-score (2.58 for a confidence level of 0.99%), ε is the margin of error, n is the sample size within an unlimited population, n′ is the finite sample size, N is the population size and Φ is the population proportion. The actual sample size was adequate for obtaining a power higher than 0.99 for all measured variables.

The Shapiro–Wilk test was used to test the normality distributions of the variables. The descriptive results were expressed as the median and interquartile range (for parameters without normal distribution), the mean ± standard deviation (for parameters with normal distribution) or as numbers and percentages (for categorical variables). Single-factor correlations between two independent variables were analysed using Pearson coefficient analysis. Linear regression was used to examine the associations between IMT or ABI and adipokines. The correlations between adipokines and the markers of subclinical atherosclerosis were further explored using binary logistic regression analysis. Receiver operating characteristics curves were used to determine the sensitivity and specificity of visfatin for predicting subclinical atherosclerosis (IMT ≥ 1 mm or plaque). Differences were considered statistically significant when they had two-sided *p*-values of less than 0.05. All statistical analyses were performed using IBM SPSS Statistics software (version 28.0.1.0).

## 3. Results

### 3.1. Characteristics of the Study Population

The clinical characteristics of the participants are shown in Table 1. After the invitation to join the study, 254 patients attended family doctor appointments. Among them, 18 eligible patients chose not to participate and 20 did not meet the inclusion criteria; therefore, 216 patients were considered eligible for the study. After statistical overviews, we removed four participants from the analysis as outliers (they had significantly higher concentrations of visfatin or resistin), which was checked using several methods, including Cook’s distance and leverage values.

### 3.2. Correlations between Adipokines and Conventional Risk Factors

We found that visfatin was positively correlated with the android to gynoid (A/G) ratio but negatively correlated with high-density lipoprotein cholesterol (Table 2). Resistin had an inverse correlation with physical activity. RBP-4 did not show any associations with the analysed factors. HMW adiponectin and leptin demonstrated significant relationships with lipid profile and body composition.

### 3.3. Correlations between Adipokines and Markers of Subclinical Atherosclerosis

Visfatin and resistin showed statistically significant correlations with atherosclerotic vascular changes, according to univariate linear and binary logistic regression analysis (Table 3).

Resistin mainly correlated with carotid atherosclerosis but failed to predict ABI or peripheral artery disease. Visfatin largely predicted IMT but did not show any correlations with plaque or peripheral artery disease.

**Figure 1:**
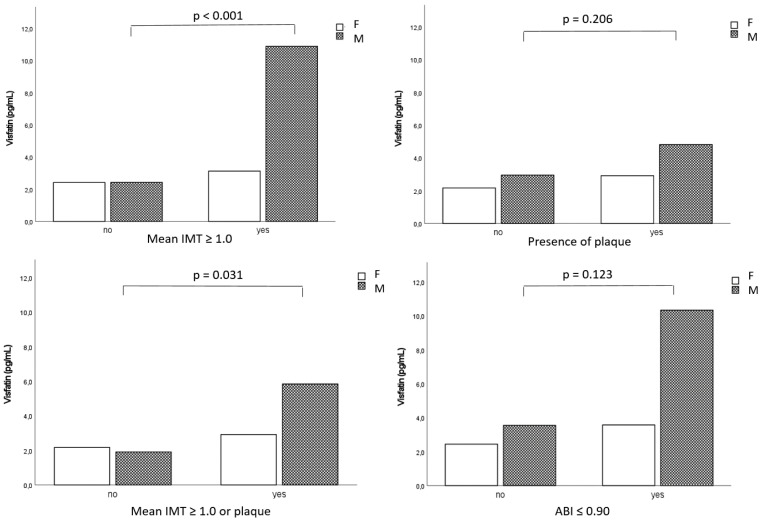
The correlations between visfatin and subclinical atherosclerosis by gender. Abbreviations: F, female; M, male; IMT, intima–media thickness; ABI, ankle–brachial index.

There was a significant difference between men and women as visfatin predicted IMT thickening in the carotid arteries of males (*p* < 0.001) but not those of females (Figure 1).

### 3.4. Effects of Medications on Visfatin and Resistin Concentrations

Without considering background treatments, statin users had lower visfatin concentrations than participants who did not use statins (2.87 ± 0.48 vs. 3.12 ± 0.47 ng/mL, respectively) and β-blocker users had higher visfatin concentrations than patients who did not use β-blockers (3.34 ± 0.62 vs. 2.88 ± 0.45 ng/mL, respectively), although these differences were not statistically significant. There were no differences in visfatin concentrations between therapy with or without CCB or ACEI/ARB. Resistin concentrations did not vary based on drug classes.

### 3.5. Effects of Medications on Correlations between Adipokines and Subclinical Atherosclerosis

**Figure 2:**
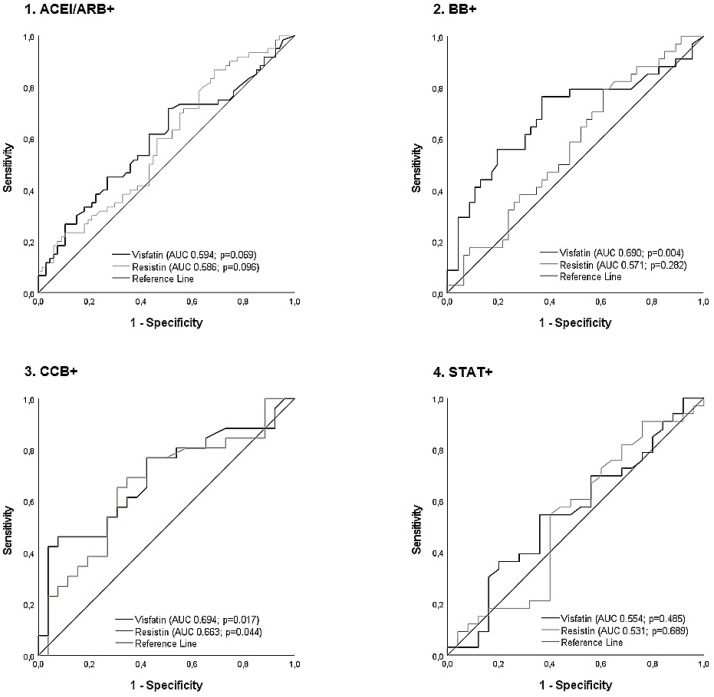
Receiver operating characteristic curve analysis for changes in the correlations between visfatin and resistin concentrations and subclinical atherosclerosis (IMT ≥ 1 or plaque) in the carotid artery by drug group, without adjustments for other medications. Abbreviations: ACEI/ARB+, angiotensin-converting enzyme inhibitors/angiotensin II receptor blockers; AUC, area under the curve; BB+, β-blocker; CCB+, calcium channel blockers; STAT+, statins.

Figure 2 shows the changes in the correlations between visfatin and resistin concentrations with IMT ≥ 1 mm or plaque in the carotid artery due to one drug class, without adjustment for others. The association between visfatin and subclinical atherosclerosis was not statistically significant among individuals using ACEI/ARB or statins.

While evaluating the individual effects of treatment groups (i.e., effects without background treatments) on visfatin concentrations and its relation to subclinical atherosclerosis, we found that patients undergoing monotherapy with statins or CCB had lower visfatin concentrations compared to the non-treatment group (2.49 ± 0.39 pg/mL and 1.91 ± 0.82 pg/mL vs. 3.18 ± 0.64 pg/mL, respectively) and patients using ACEI/ARB or β-blockers (3.37 ± 1.57 pg/mL and 3.44 ± 0.89 pg/mL, respectively). Statin monotherapy also lowered resistin concentrations compared to the other drug classes, which had levels that were similar to those in the non-treatment group (34.68 ± 13.75 ng/mL vs. 43.94 ± 18.65 ng/mL, respectively).

**Figure 3:**
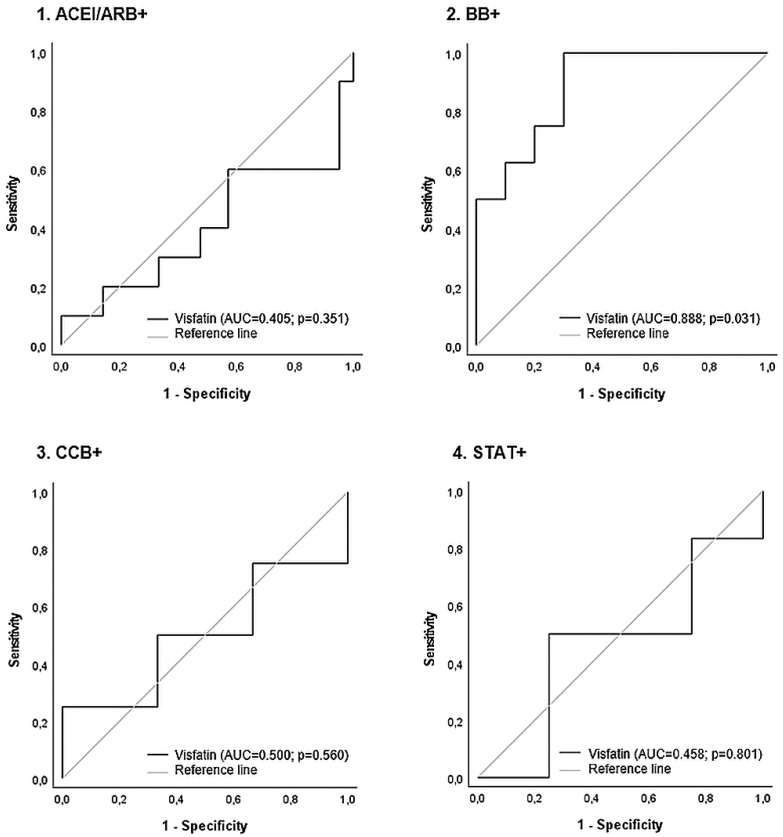
Receiver operating characteristic curve analysis for changes in the correlations between visfatin concentrations and subclinical atherosclerosis (IMT ≥ 1 or plaque) in the carotid artery, after taking into account the use of other drugs. The *p*-value was based on logistic regression analysis. Abbreviations: ACEI/ARB+, angiotensin-converting enzyme inhibitors/angiotensin II receptor blockers; AUC, area under the curve; BB+, β-blocker; CCB+, calcium channel blockers; STAT+, statins.

After carrying out receiver operating characteristics curve analysis, we established the predictive values of visfatin levels for mean IMT ≥ 1.0 mm or plaque in the carotid artery, drug by drug (Figure 3). The correlation between visfatin and subclinical atherosclerosis was statistically significant among β-blocker users but not among others. The optimal cut-off value for visfatin for the detection of subclinical atherosclerosis in patients only using β-blockers was 1.63 ng/mL, with a sensitivity of 88% and a specificity of 70% (the area under the curve was 0.888; 95% CI: 0.737–1.000; *p* = 0.006). The cut-off value for visfatin for the detection of subclinical atherosclerosis in patients using combination therapy with ACEI/ARB and CCB was 1.23 ng/mL, with a sensitivity of 86% and a specificity of 83% (the area under the curve was 0.881; 95% CI: 0.655–1.000; *p* = 0.022). The optimal cut-off value for visfatin for the detection of subclinical atherosclerosis in patients undergoing unknown treatments was 1.41 ng/mL (63% sensitivity; 60% specificity; *p* = 0.010).

### 3.6. Associations between Visfatin and Resistin and Atherosclerosis Surrogate Markers after Adjustments for Common Risk Factors

In our multiple linear regression analysis, the associations between visfatin and atherosclerosis markers (except for plaque) remained statistically significant, even after adjustments for common risk factors and the use of cardiovascular medications (Table 4). After these adjustments, resistin only correlated with mean IMT.

The model was adjusted for gender, age, hypertension (blood pressure more than 140/90 mmHg or treatment with angiotensin-converting enzyme inhibitors, angiotensin II receptor blockers, calcium channel blockers or β-blockers), hyperlipidaemia (LDL more than 2.6 mmol/L or statin therapy), the duration of diabetes, HbA1c (%), A/G ratio > 1.0, smoking habits (past and current), HDL, hsCRP and physical activity (MET-mins per week).

The model had a *p*-value of *p* < 0.001 for visfatin, except for plaque.

## 4. Discussion

We found a positive correlation between subclinical atherosclerosis and visfatin in patients with type 2 diabetes. Although visfatin concentrations were affected by β-blockers and statins, after adjustments for risk factors and cardiovascular drug use, they remained significantly correlated with carotid and peripheral atherosclerosis, particularly in male patients.

We also investigated the associations between several adipokines and subclinical atherosclerosis in patients with type 2 diabetes without known atherosclerosis. Since high molecular weight adiponectin, leptin and RBP-4 did not have statistically significant associations with subclinical atherosclerosis, they were not included in the further analysis. We primarily focused on visfatin because it had the strongest association with atherosclerosis. We also included resistin as a comparison as it showed some association with atherosclerosis.

Studies evaluating the effects of drugs on the concentrations of circulating adipokines have yielded conflicting results. This is not because of diurnal variations [19], but rather variations related to differences in study populations and/or the laboratory methods used. Commercially available enzyme-linked immunosorbent assays and enzyme or radioimmunoassay kits with different intra- and inter-assay coefficients of variance have been used [19,20,22], but there are still no universally agreed normal ranges for adipokine levels.

The pathogenesis of atherosclerosis has two distinct parts. The thickening of the intima–media and the formation of atherosclerotic plaque are biologically and genetically different entities and represent different phenotypes of atherosclerosis [30]. We found a positive correlation between visfatin and IMT, but not plaque. Several studies have suggested that visfatin contributes to atherosclerotic plaque development, progression and instability [11,12,13,30]. In most cases, these studies were conducted among patients with advanced atherosclerosis and the definitions of atherosclerosis were not consistent. Increased visfatin levels have not only been described in symptomatic rather than asymptomatic patients with carotid stenosis but also in angiographically proven stable asymptomatic coronary artery disease patients compared to healthy controls [11,12]. In these studies, plaque was defined as the localised thickening of the vessel wall of more than 2.5 mm or using the Gensini score [11,12]. Here, there were essential differences between these studies and our study with respect to the prior status of patients (i.e., without known atherosclerosis) and the definition of atherosclerosis.

Undiagnosed and untreated hypertension plays a vital role in the thickening of carotid artery intima–media. In our study, patients who were treated with ACEI/ARB or CCB lost the correlation between visfatin and IMT. This could be easily explained by the antihypertensive therapeutic effects of these drugs. However, the reported effects of antihypertensive drugs on adipokines have varied from study to study. In obese patients with essential hypertension, visfatin concentrations were not affected by candesartan or amlodipine. While resistin concentrations were significantly lower after treatment with amlodipine, no significant differences were seen after treatment with candesartan [19]. Serum visfatin levels increased with telmisartan treatment but decreased with amlodipine treatment in non-diabetic essential hypertensive patients [31]. In this study, visfatin concentrations were higher in patients with insulin resistance than those without it. This implied that visfatin is independently correlated with insulin resistance and that the effects of antihypertensive drugs on visfatin levels depend on their action on insulin resistance.

A clear difference has been described between the impacts of the ACEI and ARB drug classes. Treatment with losartan improved RBP-4 and resistin levels and decreased vascular remodelling biomarkers but elevated visfatin in hypertensive subjects, whereas ramipril had no effect [20]. There are not only differences between drug classes but also within them. Treatment with candesartan decreased RBP-4 and resistin levels and increased visfatin but no significant changes were observed with olmesartan treatment in overweight hypertensive patients with type 2 diabetes [22].

However, these studies did not assess the associations between drug-induced changes in adipokines and subclinical atherosclerosis. Based on our study, it could be concluded that high visfatin concentrations may indicate the presence of hypertension as visfatin remained an independent predictor for IMT ≥ 1 mm after adjustments for common risk factors, including antihypertension therapy. This was supported by a study by Ozal et al., who demonstrated higher visfatin levels in patients with resistant hypertension than those with controlled hypertension [32].

There is limited evidence to suggest that β-blockers affect visfatin levels in the human body. Treatment with metoprolol ameliorated decreased intracellular visfatin activity in cardiomyocytes [33], while Skoczylas et al. observed significant reductions in plasma visfatin levels compared to baseline values after 6 weeks of treatment with bisoprolol in patients with essential hypertension [19]. Contrary to previous studies and our expectations, we found that β-blocker users had higher levels of visfatin. The mechanisms by which β-blockers affect visfatin levels are not fully understood and more research is needed to clarify this relationship. It is possible that the effects of β-blockers on visfatin levels depend on the specific β -blocker, dose and/or the individual characteristics of the patient.

Statins have predictable lowering effects on visfatin concentrations. Treatment with atorvastatin, rosuvastatin and simvastatin significantly reduced visfatin levels, regardless of the selected study population or the severity of atherosclerosis [11,34,35]. The results of our study were consistent with those in the literature. Accordingly, it is essential to document the use of antidyslipidaemic drugs before attempting to interpret visfatin concentrations.

The concentrations of visfatin seem to be gender dependent. We found that male patients had higher visfatin levels, which were more strongly associated with subclinical atherosclerosis. In addition, visfatin levels were higher among males even in peripheral artery disease, although this association did not reach statistical significance. Our female patients had higher BMI values, as well as higher fat masses and lower android to gynoid fat (A/G) ratios than the males, which was more in line with expectations. Higher A/G ratios mean more visceral fat, which has known positive correlations with circulating visfatin levels [36]. However, this relationship does not depend on BMI or the overall percentage of body fat as the A/G ratio also correlates with visfatin in patients with normal weight obesity [37]. Higher concentrations of visfatin have previously been shown to be associated with obesity and insulin resistance rather than hypertension [19,31]. Despite this, our male patients were less likely to have diagnosed and treated hypertension and had higher IMT values than the women.

To the best of our knowledge, this study was the first to compare changes in resistin and visfatin concentrations according to the use of different drug classes in patients with type 2 diabetes who were not previously known to have atherosclerosis complications. The strengths of this study included the homogeneous population and the standardised conduct of the radiological procedures by a single investigator in a single centre, which minimised variations.

However, the study had some limitations. The cross-sectional and observational design did not allow us to draw firm conclusions about the influence of the drugs on visfatin levels. Causal relationships between adipokines and subclinical atherosclerosis could not be confirmed as adipokines have multicellular origins and the clinical relevance of their circulating levels must be interpreted carefully. Although the sample size was greater than those in previous studies, it was still relatively small and lacked a healthy control group. We focused on drug classes and not active substances; therefore, it was impossible to draw conclusions about specific drugs. Antidiabetic medications could also be confounding factors in modulating adipokines as they are frequently used in treatments for type 2 diabetes. Links to lower limb atherosclerosis status and peripheral artery disease were not supported by radiological imaging. However, our study raised the question of whether determining visfatin concentrations could be a cost-effective means to avoid some radiological studies in the assessment of cardiovascular risks.

Together with antihypertensive drugs and statins, antiaggregants and antidiabetics play essential roles in preventing atherosclerosis complications and their effects on adipokines deserve investigation in future studies.

## 5. Conclusions

Visfatin has significant associations with the markers of subclinical atherosclerosis in patients with type 2 diabetes, even after adjustments for common risk factors and cardiovascular medications. Therefore, measuring visfatin concentrations could aid in identifying individuals who could benefit from implementing preventive measures against atherosclerosis.

## Figures and Tables

**Table 1:** Clinical characteristics.

	All (n = 212)	Male (n = 89)	Female (n = 123)	*p*-Value
Age	58.96 ± 8.02	56.38 ± 9.02	60.78 ± 6.66	<0.001
Duration of DM	7.07± 5.99	6.45 ± 5.51	7.52 ± 6.29	NS
Body Composition				
Weight	96.69 ± 1.18	103.17 ± 16.08	92.00 ± 16.58	<0.001
BMI	34.07 ± 5.74	32.83 ± 4.87	34.96 ± 6.17	0.007
VAI	2.12 (1.86)	2.2 (2.09)	2.06 (1.81)	NS
A/G (%) Ratio	1.23 ± 0.23	1.39 ± 0.29	1.12 ± 0.17	<0.001
Arm Fat Mass (kg)	3.78 ± 1.33	3.09 ± 1.14	4.29 ± 1.22	<0.001
Leg Fat Mass (kg)	10.63 ± 4.46	8.12 ± 3.08	12.46 ± 4.43	<0.001
Trunk Fat Mass (kg)	21.56 ± 5.70	21.37 ± 6.18	21.7 ± 5.34	NS
Android Fat Mass (kg)	3.98 ± 1.20	4.02 ± 1.29	3.95 ± 1.14	NS
Gynoid Fat Mass (kg)	5.47 ± 1.85	4.57 ± 1.56	6.12 ± 1.77	<0.001
Total Fat Mass (kg)	36.97 ± 10.53	33.51 ± 10.07	39.5 ± 10.16	<0.001
Total Fat (%)	39.4 ± 8.1	33.0 ± 6.27	44.14 ± 5.66	<0.001
Additional Diagnoses				
Hypertension	185 (87.3%)	73 (82.0%)	112 (91.1%)	0.052
Hyperlipidaemia	109 (51.4%)	45 (50.6%)	64 (52.0%)	NS
Systolic (mmHg)	145.98 ± 17.81	145.63 ± 17.59	146.24 ± 18.03	NS
Diastolic (mmHg)	88.66 ± 9.22	89.82 ± 9.20	87.82 ± 9.18	NS
Laboratory Methods				
HbA1c (%)	6.88 ± 1.19	7.04 ± 1.38	6.77 ± 1.03	NS
hsCRP (mg/mL)	3.90 ± 5.14	3.61 ± 5.13	4.11 ± 5.15	NS
LDL (mmol/L)	3.48 ± 1.17	3.45 ± 1.19	3.50 ± 1.16	NS
HDL (mmol/L)	1.36 ± 0.34	1.24 ± 0.32	1.45 ± 0.33	<0.001
TG (mmol/L)	2.14 ± 1.75	2.44 ± 2.24	1.92 ± 1.24	0.034
Total C (mmol/L)	5.58 ± 1.26	5.54 ± 1.25	5.61 ± 1.27	NS
Non-HDL (mmol/L)	4.2 ± 1.22	4.3 ± 1.22	4.15 ± 1.23	NS
HMW Adiponectin (µg/mL)	3.36 ± 2.50	2.49 ± 2.05	3.99 ± 2.62	<0.001
Leptin (ng/mL)	19.59 (21.50)	12.52 (12.62)	28.72 (23.29)	<0.001
Resistin (ng/mL)	42.82 ± 18.21	39.76 ± 16.89	45.05 ± 18.88	0.034
RBP-4 (µg/mL)	72.09 (30.31)	71.84 (34.24)	72.3 (29.15)	NS
Visfatin (pg/mL)	3.05 (0.38)	3.85 (0.77)	2.48 (0.28)	NS
Therapy				
Statins	58 (27.4%)	25 (28.1%)	33 (26.8%)	NS
ACEI or ARB	127 (59.9%)	42 (47.2%)	85 (69.1%)	0.001
β-Blockers	80 (37.7%)	25 (28.1%)	55 (44.7%)	0.014
CCB	52 (24.5%)	17 (19.1%)	35 (28.5%)	NS
Antiaggregant	52 (24.5%)	24 (27.0%)	28 (22.8%)	NS
Metformin	178 (84.0%)	75 (84.3%)	103 (83.7%)	NS
GLP-1 RA	5 (2.4%)	1 (1.1%)	4 (3.3%)	NS
SLT-2I	6 (2.8%)	4 (4.5%)	2 (1.6%)	NS
DPP-4I	35 (16.5%)	18	17 (13.8%)	NS
TZD	2 (0.9%)	1 (1.1%)	1 (0.8%)	NS
SU	62 (29.2%)	29 (32.6%)	33 (26.8%)	NS
Insulin	20 (9.4%)	12 (13.5%)	8 (6.5%)	NS
Oral Antidiabetic Therapy	185 (87.3%)	78 (87.6%)	107 (87.0%)	NS
Antihypertensive Therapy	159 (75.0%)	56 (62.9%)	103 (83.7%)	<0.001
None	8 (3.8%)	3 (3.4%)	5 (4.1%)	NS
Smoking				
Never	119 (56.1%)	29 (32.6%)	90 (73.2%)	<0.001
Past	52 (24.5%)	40 (44.9%)	12 (9.8%)	<0.001
Current	41 (19.3%)	20 (22.5%)	21 (17.1%)	NS
Physical Activity				
(MET-mins per week)	4634.13 ± 270.44	5567.40 ± 452.73	3958.84 ± 319.60	0.003
Subclinical Atherosclerosis				
IMT (mm)	0.83 ± 0.01	0.85 ± 0.01	0.82 ± 0.01	0.047
IMT ≥ 1.0	55 (25.9%)	24 (27.0%)	31 (25.2%)	NS
Presence of Plaque	94 (44.3%)	43 (48.3%)	51 (41.5%)	NS
ABI	1.09 ± 0.01	1.11 ± 0.02	1.07 ± 0.01	0.042
ABI < 0.9	8 (3.8%)	4 (4.5%)	4 (3.3%)	NS

Data are expressed as mean ± SD, median and IQR or n and %. Abbreviations: DM, diabetes mellitus; BMI, body mass index; VAI, visceral adiposity index; A/G, android to gynoid ratio; HbA1c, haemoglobin A1C; hsCRP, high-sensitivity C-reactive protein; LDL, low-density lipoprotein; HDL, high-density lipoprotein; TG, triglycerides; Total C, total cholesterol; RBP-4, retinol-binding protein 4; HMW, high molecular weight; ACEI, angiotensin-converting enzyme inhibitors; ARB, angiotensin II receptor blockers; CCB, calcium channel blockers; GLP-1 RA, glucagon-like peptide-1 receptor agonists; SLGT2I, sodium-glucose co-transporter-2 inhibitors; DPP4I, dipeptidyl peptidase-4 inhibitors; TZD, thiazolidinediones; SU, sulphonylureas; MET-mins, metabolic equivalent of task minutes; IMT, intima–media thickness; ABI, ankle–brachial index.

**Table 2:** Correlations between adipokines and conventional cardiovascular risk factors.

		HMW Adiponectin	Leptin	Resistin	RBP-4	Visfatin
SBP	r	-	-	-	-	-
	*p*	NS	NS	NS	NS	NS
DBP	r	-	-	-	-	-
	*p*	NS	NS	NS	NS	NS
Duration of DM	r	-	-	-	-	-
	*p*	NS	NS	NS	NS	NS
Total C	r	0.152	-	-	-	-
	*p*	0.027	NS	NS	NS	NS
HDL	r	0.545	0.151	-	-	−0.175
	*p*	<0.001	0.027	NS	NS	0.011
LDL	r	-	-	-	-	-
	*p*	NS	NS	NS	NS	NS
TG	r	−0.25	-	-	-	-
	*p*	<0.001	NS	NS	NS	NS
Non-HDL	r	-	-	-	-	-
	*p*	NS	NS	NS	NS	NS
HbA1c	r	−0.267	-	-	-	-
	*p*	<0.001	NS	NS	NS	NS
BMI	r	−0.15	0.503	-	-	-
	*p*	0.029	<0.001	NS	NS	NS
VAI	r	−0.199	-	-	-	-
	*p*	0.004	NS	NS	NS	NS
A/G ratio	r	−0.397	−0.33	-	-	0.153
	*p*	<0.001	<0.001	NS	NS	0.026
Age	r	0.181	0.225	-	-	-
	*p*	0.008	0.001	NS	NS	NS
MET-mins	r	-	−0.237	−0.14	-	-
	*p*	NS	0.001	0.042	NS	NS

Abbreviations: r, Pearson’s r; *p*, *p*-value; NS, non-significant; HMW, high molecular weight; RBP-4, retinol-binding protein 4; SBP, systolic blood pressure; DPB, diastolic blood pressure; DM, diabetes mellitus; Total C, total cholesterol; HDL, high-density lipoprotein; LDL, low-density lipoprotein; TG, triglycerides; HbA1c, haemoglobin A1C; BMI, body mass index; VAI, visceral adiposity index; A/G, android to gynoid ratio; MET-mins, metabolic equivalent of task minutes.

**Table 3:** Correlations between adipokines and subclinical atherosclerosis.

		IMT	ABI	IMT ≥ 1.0	Plaque	IMT ≥ 1.0 or Plaque	ABI ≤ 0.9
HMW Adiponectin	β/EXP(B)	-	-	-	-	-	-
	*p*	NS	NS	NS	NS	NS	NS
Leptin	β/EXP(B)	-	-	-	-	-	-
	*p*	NS	NS	NS	NS	NS	NS
Resistin	β/EXP(B)	0.179	-	-	1.000	-	-
	*p*	0.009	NS	NS	0.049	NS	NS
RBP-4	β/EXP(B)	-	-	-	-	-	-
	*p*	NS	NS	NS	NS	NS	NS
Visfatin	β/EXP(B)	0.212	−0.141	1.139	-	1.136	-
	*p*	0.002	0.040	0.002	NS	0.008	NS

Linear or logistic regression analysis (as appropriate). Abbreviations: IMT, intima–media thickness; ABI, ankle–brachial index; HMW, high molecular weight; *p*, *p*-value; NS, non-significant; RBP-4, retinol-binding protein 4.

**Table 4:** Associations between subclinical atherosclerosis and visfatin and resistin.

		Visfatin			Resistin		
		β	t/Exp(B)	*p*	β	t/Exp(B)	*p*
Mean IMT		0.206	3.06	0.003	0.137	1.99	0.049
ABI		−0.151	−2.09	0.038	-	-	NS
IMT > 1.0		0.136	1.146	0.006	-	-	NS
Plaque		-	-	NS	-	-	NS
IMT > 1.0 or Plaque		0.160	1.174	0.005	-	-	NS
ABI ≤ 0.9		0.107	1.113	0.029	-	-	NS

Linear or logistic regression analysis (as appropriate). Abbreviations: IMT, intima–media thickness; ABI, ankle–brachial index; *p*, *p*-value; NS, non-significant.

## Data Availability

The data described in the manuscript are available upon reasonable request.
